# Membrane curvature sensing and stabilization by the autophagic LC3 lipidation machinery

**DOI:** 10.1126/sciadv.add1436

**Published:** 2022-12-14

**Authors:** Liv E. Jensen, Shanlin Rao, Martina Schuschnig, A. King Cada, Sascha Martens, Gerhard Hummer, James H. Hurley

**Affiliations:** ^1^ Department of Molecular and Cell Biology, University of California Berkeley, Berkeley, CA 94720, USA.; ^2^ California Institute for Quantitative Biosciences, University of California, Berkeley, CA 94720, USA.; ^3^ Aligning Science Across Parkinson’s (ASAP) Collaborative Research Network, Chevy Chase, MD 20815, USA.; ^4^ Department of Theoretical Biophysics, Max Planck Institute of Biophysics, Frankfurt am Main, Germany.; ^5^ Department of Biochemistry and Cell Biology, Max Perutz Labs, University of Vienna, Vienna BioCenter, Vienna, Austria.; ^6^ Institute of Biophysics, Goethe University Frankfurt, Frankfurt am Main 60438, Germany.; ^7^ Helen Wills Neuroscience Institute, University of California, Berkeley, Berkeley, CA 94720, USA.

## Abstract

How the highly curved phagophore membrane is stabilized during autophagy initiation is a major open question in autophagosome biogenesis. Here, we use in vitro reconstitution on membrane nanotubes and molecular dynamics simulations to investigate how core autophagy proteins in the LC3 (Microtubule-associated proteins 1A/1B light chain 3) lipidation cascade interact with curved membranes, providing insight into their possible roles in regulating membrane shape during autophagosome biogenesis. ATG12(Autophagy-related 12)–ATG5-ATG16L1 was up to 100-fold enriched on highly curved nanotubes relative to flat membranes. At high surface density, ATG12–ATG5-ATG16L1 binding increased the curvature of the nanotubes. While WIPI2 (WD repeat domain phosphoinositide-interacting protein 2) binding directs membrane recruitment, the amphipathic helix α2 of ATG16L1 is responsible for curvature sensitivity. Molecular dynamics simulations revealed that helix α2 of ATG16L1 inserts shallowly into the membrane, explaining its curvature-sensitive binding to the membrane. These observations show how the binding of the ATG12–ATG5-ATG16L1 complex to the early phagophore rim could stabilize membrane curvature and facilitate autophagosome growth.

## Teaser

Components of the LC3 lipidation machinery sense membrane curvature, pointing to a potential role in shaping autophagosomes.

## INTRODUCTION

Macroautophagy, hereafter autophagy, is the process in which cytosolic cargoes such as protein aggregates, damaged organelles, intracellular pathogens, or bulk cytoplasm are engulfed in a double-membrane vesicle and targeted to the lysosome for degradation (*1*). Autophagic dysfunction results in defects in the clearance of aggregates and damaged organelles, contributing to human neurodegenerative diseases, including Parkinson's disease (*2*, *3*). During cargo engulfment, the cup-shaped phagophore grows progressively larger (*4*) and ultimately closes into the double-membraned autophagosome. The past few years have brought rapid progress in understanding how autophagy is initiated, cargo is selected, and lipids are sourced and transferred for autophagosome expansion (*5*–*7*). In this context, the physical mechanism of membrane shaping and stabilization during autophagosome growth remains one of the most prominent open questions.

The formation and stabilization of the cup-shaped membrane during phagophore expansion are associated with energetic penalties and barriers (*8*–*10*). Recent cryo–electron tomography visualization of phagophores in yeast revealed that the membrane curvature at the phagophore rim approaches the maximum value possible given the thickness of the phospholipid bilayer (*11*). The recruitment of membrane curvature–inducing proteins to the phagophore rim is currently a leading model to explain how its high curvature is stabilized (*9*). A number of core autophagy proteins have been shown to sense membrane curvature (*12*). The class III phosphatidylinositol 3-kinase complex I (PI3KC3-C1) subunit ATG14 senses membrane curvature through its C-terminal BATS (Barkor/Atg14(L) autophagosome targeting sequence) domain (*13*–*15*). Both the human phospholipid transporter ATG2A (*9*) and the *Arabidopsis* ATG5 subunit of the ATG12–ATG5-ATG16L1 complex (*16*) localize to toroidal structures that appear to correspond to the phagophore rim. ATG3 (*17*) and the ATG12–ATG5-ATG16L1 complex (*18*), which are both involved in the conjugation of ATG8 proteins to membranes, preferentially bind to small liposomes through amphipathic helices at or near their N termini. Thus, both cell imaging (*16*) and in vitro binding (*18*) data suggested that the ATG12–ATG5-ATG16L1 complex could stabilize the phagophore rim via a preference for high curvature membrane binding.

**Fig. 1.**
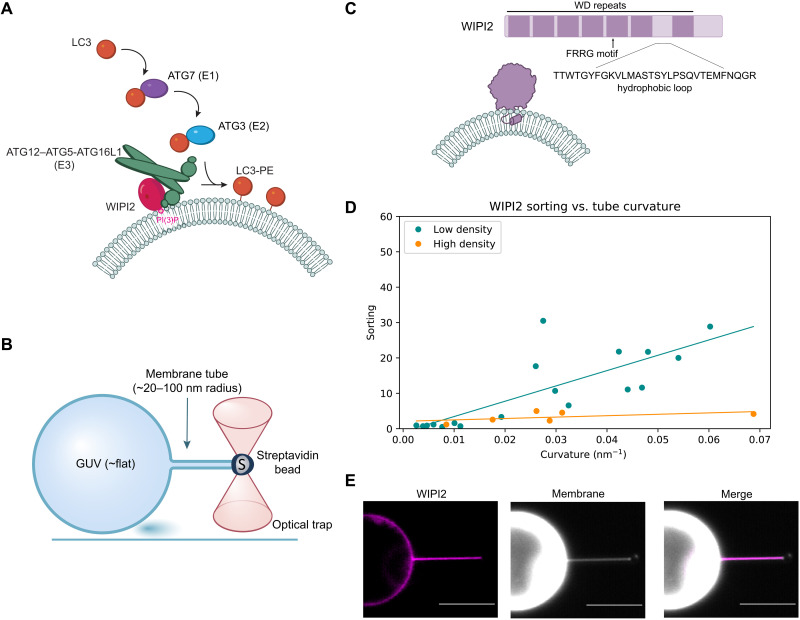
WIPI2 senses membrane curvature. (**A**) Overview of LC3 lipidation machinery at a phospholipid bilayer. (**B**) Schematic of GUV/optical trap setup for quantification of membrane curvature sensing. (**C**) Graphical representation of WIPI2 domain architecture and interaction with curved membrane. (**D**) Plot of WIPI2 sorting versus membrane tube curvature at high (*n* = 6, *r*^2^ = 0.30) and low (*n* = 18, *r*^2^ = 0.64) surface densities of WIPI2 on the GUV surface. Each data point represents an individual membrane tube. (**E**) Representative confocal fluorescence microscopy images of WIPI2 localization on membrane tube and GUV surface. Scale bars, 10 μm.

Covalent conjugation of the ATG8 family proteins LC3A-C, GABARAP (Gamma-aminobutyric acid receptor-associated protein), and GABARAPL1/2 to the membrane lipid phosphatidylethanolamine (PE), hereafter referred to as “LC3 lipidation,” contributes to phagophore expansion (*19*) and the recruitment of cargo and other autophagy proteins (*20*, *21*). LC3 lipidation proceeds through a cascade of enzymes analogous to the ubiquitin E1/E2/E3 ligase mechanism (Fig. 1A). The E1 ATG7 binds LC3, handing it off to the E2 ATG3, which works together with the E3 ATG12–ATG5-ATG16L1 to covalently conjugate LC3 to PE headgroups on the phagophore. The ATG12–ATG5-ATG16L1 is recruited to phosphatidylinositol 3-phosphate (PI(3)P)-positive autophagic membranes by the PROPPIN WIPI2 through its binding to a WIPI2 interacting region in ATG16L1 (*22*, *23*). These reactions have been reconstituted in vitro on sonicated liposomes (*17*, *24*) and flat membranes (*25*). In this study, we sought to systematically examine the impact of curvature on this machinery using a precisely tunable and quantitative in vitro system and to gain a detailed molecular view of the membrane interactions with the help of molecular dynamics (MD) simulations.

Optical tweezers can be used to pull membrane nanotubes from a giant unilamellar vesicle (GUV) and form a contiguous membrane system with regions of high and low curvature, enabling quantitation of the curvature sensitivity of proteins (Fig. 1B) (*26*, *27*). This experimental setup can access nanoscale dimensions similar to the curvature of the phagophore rim [<30 nm (*11*)]. The physics of membrane nanotubes pulled under force from an optical trap allows their radius to be measured by fluorescence microscopy even for dimensions far below the optical diffraction limit. Here, we used this approach to characterize the WIPI2 and ATG12–ATG5-ATG16L1 system and find that it is profoundly curvature sensitive.

## RESULTS

### WIPI2 interactions with membrane tubes

WIPI2 is responsible for the membrane recruitment of ATG12–ATG5-ATG16L1 in cells (*22*, *23*), so we began by assessing how WIPI2 interacts with membranes of varying curvature. WIPI2 is a member of the PROPPIN family of proteins, which bind membranes through a hydrophobic loop (*28*) together with an FRRG motif that specifically recognizes PI(3)P (Fig. 1C) (*28*–*31*). We used the membrane nanotube assay to measure the curvature sensitivity of WIPI2 by visualizing WIPI2 localization to highly curved membrane tubes and the essentially flat GUV from which they were pulled (Fig. 1D). PI(3)P-positive GUVs were incubated with fluorescently labeled mCherry-WIPI2, and the curvature sensitivity of WIPI2 was assessed by its sorting ratio [*S*, the ratio of protein surface density on the tube to the protein density on the GUV surface (*26*)]. At the limit of low density, the sorting ratio describes the tendency of membrane-bound proteins to preferentially associate with the curved tube compared to the molecularly flat GUV surface.

Analysis of WIPI2 localization showed that it preferentially binds to membrane tubes compared to the GUV surface and more strongly sorts onto narrow tubes compared to wide tubes. The sorting dependence on curvature for WIPI2 is most apparent on GUVs with a low protein surface density (ϕ_v_), reaching *S*_w_ = 14 ± 5 for tubes with radii between 20 and 40 nm (Fig. 1, D and E). As expected, the sorting ratio of WIPI2 diminishes with increasing protein surface density on the GUV, which is attributable to approaching surface area saturation on the tube (*27*, *32*). A linear fit shows a monotonic increase in sorting with curvature, similar to proteins that are known to insert into lipid bilayers via amphipathic helices (*33*).

To test whether the observed curvature-dependent sorting of WIPI2 is intrinsic to the protein itself and not due to enrichment of PI(3)P on the membrane tubes, we analyzed the localization of a fluorescently labeled PI(3)P probe, mCherry-Hrs (hepatocyte growth factor-regulated tyrosine kinase substrate) FYVE (Fab1, YOTB, Vac1, and EEA1). We found that, in contrast to WIPI2, mCherry-FYVE exhibited no enrichment on membrane tubes compared to the GUV surface (fig. S1A), indicating that sorting of WIPI2 is due to its inherent membrane curvature sensing activity.

### ATG12–ATG5-ATG16L1 has enhanced curvature sorting

**Fig. 2.**
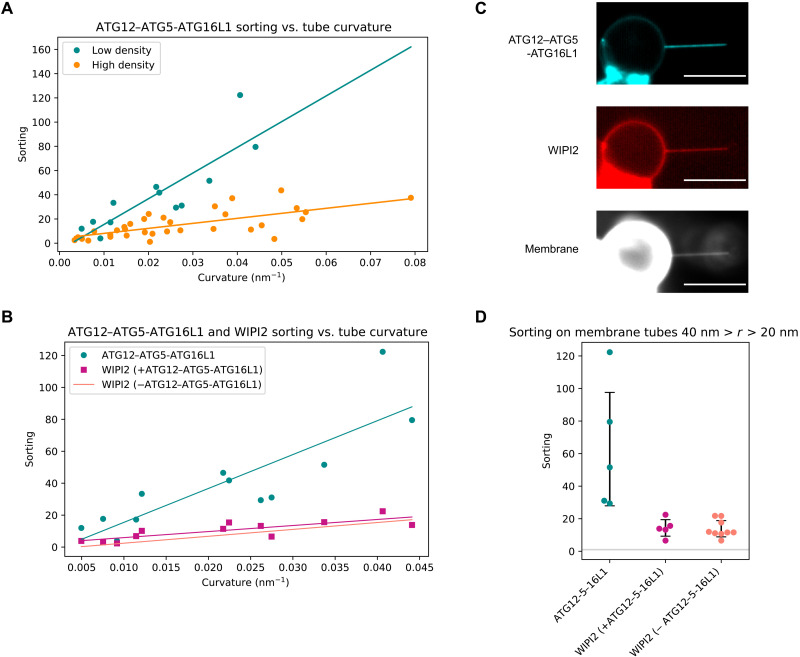
ATG12–ATG5-ATG16L1 has enhanced curvature sensitivity compared to WIPI2. (**A**) Quantification of ATG12–ATG5-ATG16L1 sorting onto membrane tubes at high (cyan, *n* = 12, *r*^2^ = 0.47) and low (orange, *n* = 33, *r*^2^ = 0.72) membrane surface densities of protein. Each data point represents an individual membrane tube. (**B**) Comparison of sorting dependence on curvature for ATG12–ATG5-ATG16L1 (cyan) and WIPI2 (magenta, *r*^2^ = 0.66) with two-color fluorescence imaging compared to sorting of WIPI2 in the absence of ATG12–ATG5-ATG16L1 (salmon). Scale bars, 10 μm. (**C**) Representative image of membrane tube enrichment of ATG12–ATG5-ATG16L1 and WIPI2 from (A) and (B). (**D**) Swarmplot depicting the sorting of ATG12–ATG5-ATG16L1 and WIPI2 (*n* = 5), compared to WIPI2 in the absence of ATG12–ATG5-ATG16L1 (*n* = 9) on membrane tubes with radii between 20 and 40 nm, black bars indicate SD, and horizontal gray line at *S* = 1.

Because both WIPI2 and ATG16L1 contain membrane binding motifs and because WIPI2 is required to recruit ATG12–ATG5-ATG16L1 to flat membranes (*25*), we sought to understand the relative roles of WIPI2 and ATG16L1. We pulled membrane nanotubes from GUVs incubated with mCherry-WIPI2 and ATG12–ATG5-ATG16L1–green fluorescent protein (GFP) and analyzed their respective recruitment to the membrane tube (Fig. 2, A to C). Just as for WIPI2, the sorting index *S* of ATG12–ATG5-ATG16L1 depends on protein surface density. Lower protein density on the GUV correlated with strong sorting of ATG12–ATG5-ATG16L1 onto membrane tubes (Fig. 2A). ATG12–ATG5-ATG16L1 was more strongly enriched on membrane tubes than WIPI2, with a sorting ratio *S*_E3_ = 63 ± 35 for membrane tubes with radii between 20 and 40 nm, compared to a sorting ratio *S*_W_ = 15 ± 4 for WIPI2 (Fig. 2, B and D).

### ATG16L1 and WIPI2 curvature sensing is independent of ATG3

Having established the potent membrane curvature sensitivity of WIPI2 and ATG12–ATG5-ATG16L1, we next tested their curvature sensitivity in the presence of ATG3, the LC3 lipidation E2 enzyme. Previously identified as a membrane curvature sensor through an N-terminal amphipathic helix (*17*), we hypothesized that ATG3 might be able to even further increase the curvature-dependent sorting of WIPI2 or ATG12–ATG5-ATG16L1 through a direct interaction.

**Fig. 3.**
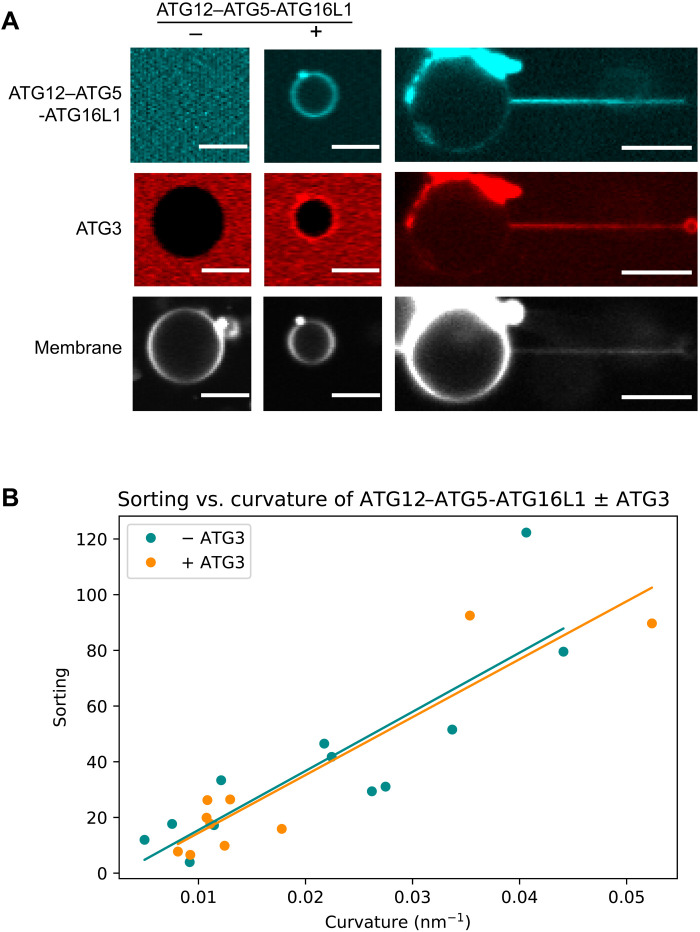
ATG12–ATG5-ATG16L1 senses curvature independently from ATG3. (**A**) ATG3 localization to GUVs and membrane tubes in the presence and absence of ATG12–ATG5-ATG16L1. Scale bars, 10 μm. (**B**) Curvature dependence of ATG12–ATG5-ATG16L1 sorting on membrane tubes in the presence (*n* = 9, *r*^2^ = 0.86) and absence (*n* = 12, from Fig. 2A, *r*^2^ = 0.72) of ATG3. Each data point represents an individual membrane tube.

First, we tested the interaction between ATG3 and ATG12–ATG5-ATG16L1. ATTO565-labeled ATG3 was incubated with GUVs alone or in the presence of unlabeled WIPI2 and ATG12–ATG5-ATG16L1-GFP. ATG3 was only recruited to the GUV surface in the presence of ATG12–ATG5-ATG16L1 and copartitioned with ATG12–ATG5-ATG16L1 onto membrane tubes (Fig. 3A). To assess the impact of ATG3 on the curvature sensitivity of ATG12–ATG5-ATG16L1 while controlling for potential differences in sorting induced by the fluorescent tags, we incubated GFP-labeled ATG12–ATG5-ATG16L1 with unlabeled ATG3 and mCherry-WIPI2. We found that the addition of ATG3 did not alter the partitioning of ATG12–ATG5-ATG16L1 onto membrane tubes compared to ATG12–ATG5-ATG16L1 in the presence of WIPI2 alone (Fig. 3B). Despite the importance of its amphipathic helix for LC lipidation activity (*17*), ATG3 did not measurably augment the curvature sensitivity of WIPI2 and ATG12–ATG5-ATG16L1.

### ATG16L1 helix α2 is responsible for curvature sensitivity

We next sought to understand the mechanism for the potent curvature sensitivity of the ATG12–ATG5-ATG16L1 complex. A previous report had characterized the membrane interactions of amphipathic helix α2, located near the N terminus of ATG16L1 (*18*). On highly curved sonicated vesicles, the physiological requirement for WIPI2 membrane recruitment can be bypassed (*25*). Helix α2 was shown to be essential for WIPI2-independent activity of ATG12–ATG5-ATG16L1 on sonicated liposomes and essential for LC3 lipidation and autophagic flux in both bulk and selective autophagy in cells (*18*). We use the sameF32A/I35A/I36A mutation, hereafter "FII mutation," previously shown to block autophagy and LC3 lipidation in cells and assessed its impact on curvature sorting of ATG12–ATG5-ATG16L1.

**Fig. 4.**
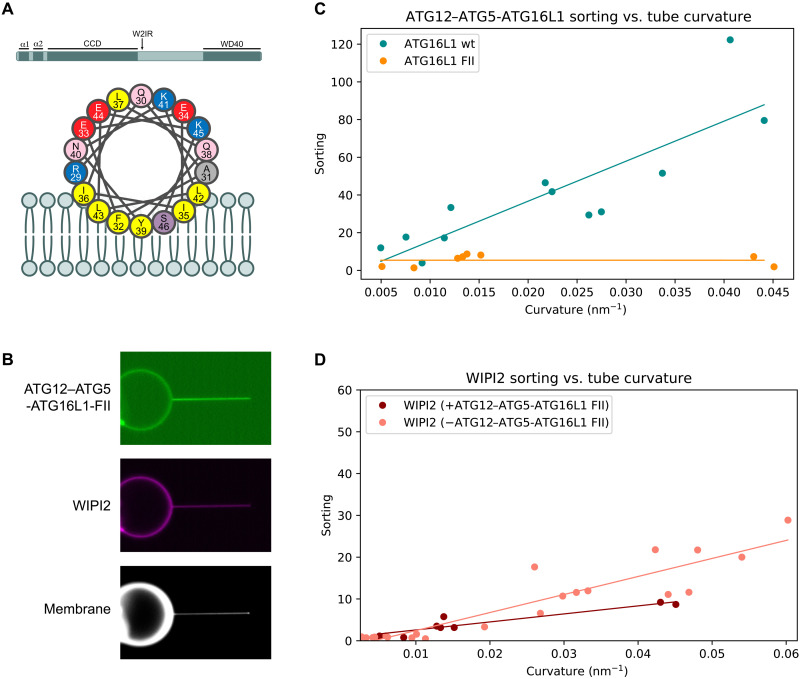
Mutagenesis affects ATG12–ATG5-ATG16L1 curvature sensitivity. (**A**) Graphical representation of the domain architecture of ATG16L1 and of the α2 membrane binding amphipathic helix. (**B**) Representative fluorescence microscopy image of E3-FII and WIPI2 localization on GUV and membrane tube surface. (**C**) Sorting versus curvature plot for ATG12–ATG5-ATG16L1 wt (*n* = 12, from Fig. 2A) compared to FII mutant (*n* = 8). (**D**) Sorting versus curvature plot for WIPI2 alone (*n* = 18, from Fig. 1D) or in the presence of ATG12–ATG5-ATG16L1 FII mutant (*n* = 8).

A GFP-tagged version of the previously described FII mutant of ATG16L1 was purified and incubated with GUVs in the presence of mCherry-WIPI2 (Fig. 4, A and B). The FII mutation was sufficient to ablate the curvature sensitivity of ATG12–5-16L and caused a minor decrease in the sorting of WIPI2 onto membrane tubes (Fig. 4, C and D). We conclude that the molecular basis for the amplified curvature-dependent sorting we observed for ATG16L1 depends on hydrophobic residues in the amphipathic helix α2 of ATG16L1.

### The ATG12–ATG5-ATG16L1 complex induces membrane curvature at high surface density

**Fig. 5.**
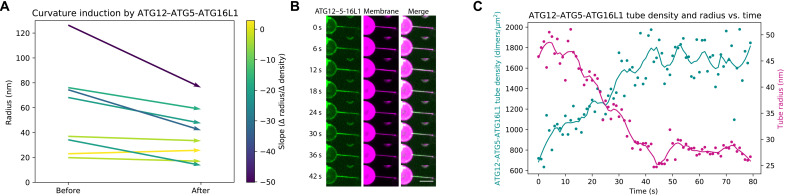
Curvature induction by ATG12–ATG5-ATG16L1. (**A**) Initial and final tube radii after the accumulation of ATG12–ATG5-ATG16L1 onto the membrane tube. Vectors color-coded for slope of the change in protein density over the change in tube radius, where darker colors indicate a steeper negative slope. (**B**) Montage of a single representative membrane tube showing ATG12–ATG5-ATG16L1 localization relative to the tube and membrane tube intensity over time. Snapshots of a continuous acquisition every 6 s, showing ATG16L1-GFP (green) and ATTO647N-DOPE membrane (magenta). Scale bars, 10 μm. (**C**) Quantification of protein binding and membrane tube radius from time course in (B). Surface density of ATG16L1 dimers on the membrane tube (cyan) and membrane tube radius (magenta) plotted against time (s) for an 80-s time course.

Thermodynamic principles of curvature sensing proteins dictate that at sufficiently high protein density, a curvature sensor becomes a curvature inducer (*32*). Therefore, we wondered whether ATG12–ATG5-ATG16L1 could induce membrane curvature at high surface densities at a physiologically plausible bulk concentration (100 nM). We analyzed time-course images from nanotubes incubated with mCherry-WIPI2 and ATG12–ATG5-ATG16L1–GFP (Fig. 2), assessing how membrane tube radius changed with increasing protein surface density on the membrane tube. In these experiments, protein is preincubated with GUVs before the membrane tube is formed; after pulling the membrane tube, protein diffuses onto the tube from the GUV until it reaches its equilibrium sorting value. Taking advantage of the changing protein surface density, we measured the tube radius before and after protein enrichment on the membrane tube. Enrichment of ATG12–ATG5-ATG16L1 on membrane tubes consistently correlated with a concomitant decrease in tube radius (Fig. 5A). Visualizing a single representative tube over the course of several minutes shows that protein enrichment on the tube from ~600 to ~1700 dimers/μm^2^ induces an increase in curvature from 50 to 25 nm radius, as calculated from the decrease in the fluorescence signal from the membrane label in the tube (Fig. 5, B and C). A decrease in fluorescence was not observed on tubes with already high curvature (Fig. 5A).

### MD simulations of curved membrane binding

**Fig. 6.**
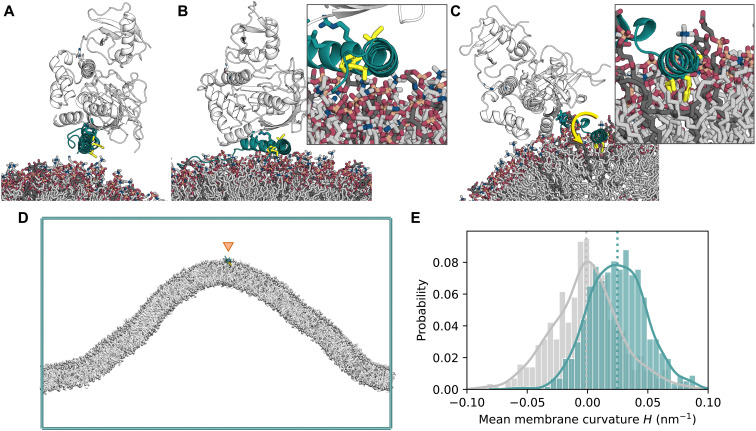
ATG16L1 helix α2 shows preference for positive curvature in MD simulations. (**A**) Initial structure (PDB ID: 4NAW) of ATG16L1 helix α2 (cyan) in complex with ATG12-5 (white) near the membrane. The hydrophobic-face residues Phe^32^, Ile^35^, and Ile^36^ are highlighted as yellow sticks. Negatively charged lipids [PI(3)P, POPI, and DOPS] are shown as dark gray sticks, with neutral lipids (DOPC and DOPE) in lighter gray. (**B**) Snapshot of ATG1612–5-L1 interacting with the membrane surface during one (1 μs) simulation replicate, with rotation of the hydrophobic face by ~180°. (**C**) End frame of a 300-ns replicate with helix α2 embedded into the membrane. (**D**) End frame of a 50-μs coarse-grained curvature sampling simulation replicate with ATG16L1 helix α2 (residues 26 to 45; located at the positively curved cusp of the membrane fold). (**E**) Probability histogram of local mean curvature values sampled at the center of mass of helix α2 (cyan) and at random lipid phosphate positions (gray) during the same simulations, with data collected over six independent 50-μs replicates, after an equilibration period of 5 μs upon each membrane binding event.

To clarify the mechanism of membrane association and curvature sensing by ATG16L1, we performed MD simulations. Structural work has shown how the N terminus of ATG16L1 interacts with the other subunits of the ATG12–ATG5-ATG16L1 complex via helix α1 (*34*). In atomistic MD simulations of helix α2 of ATG16L1 as bound to ATG12-5 near negatively charged membranes (Fig. 6A), membrane association of ATG16L1 occurred within the first few nanoseconds of each 1-μs replicate. A stable interaction interface was maintained between α1 and ATG5 (fig. S2A). Helix α2 exhibited considerable flexibility relative to α1, with swinging and rotation about the hinge region around Gln^30^ and Ala^31^. This led to spontaneous reorientation (by up to ~190° relative to the initial conformation; fig. S2B) of the hydrophobic face of α2, with the side chains of Phe^32^ and Ile^36^ brought to the protein-membrane interface (Fig. 6B) in two of five replicates (2 of the 10 ATG16L1 molecules simulated). These observations are consistent with membrane binding by the N-terminal region of ATG16L1 via exposure and insertion of hydrophobic side chains of α2. However, although the hydrophobic residues formed contacts with the lipids, they did not insert into the headgroup region of the bilayer on the time scale of the simulations. Instead, helices α1 and α2 of ATG16L1 remained above the membrane and formed primarily electrostatic interactions of their basic residues, frequently involving negatively charged lipids (Fig. 6B).

To examine the membrane-binding configuration of ATG12–ATG5-ATG16L1 brought about by rotation of α2, a separate set of simulations was initiated using a remodeled structure of ATG16L1. Here, the hydrophobic face of α2 was embedded into the membrane before simulation (Fig. 6C). Over a total simulation time of ~1.8 μs across six independent copies of the molecule, the side chains of Phe^32^, Ile^35^, and Ile^36^ remained stably embedded within the membrane (fig. S2C) and maintained interactions with lipid acyl tails, while α1 mediated contact with ATG12-5 above the membrane (Fig. 6C).

Amphipathic helix motifs have previously been shown to recognize packing defects in membranes, which increase with increasing membrane curvature (*35*–*40*). The curvature preference of helix α2 was assessed using coarse-grained MD simulations of the isolated ATG16L1 construct (residues 26 to 45) on buckled membranes (Fig. 6D) (*41*). In these longer (50 μs) simulations, multiple membrane insertion and dissociation events were observed on a microsecond time scale. During its periods of membrane association, α2 diffused within the buckled membrane and showed a strong preference for regions with high positive local curvature. Sampling at the center of mass of α2 (and allowing for an equilibration period for the helix to reach its preferred membrane regions), the distribution of local mean curvature *H* of the membrane profile showed a clear shift toward positive curvature relative to random samples of lipid headgroup positions, with a mean value of *H* ~ 0.025 nm^−1^ (Fig. 6E).

Residues 272 to 296 within the 6CD loop of WIPI2d (*23*) were identified to constitute a candidate curvature-sensing element based on secondary structure prediction (*42*), analysis of physicochemical properties (*43*), and atomistic MD simulations. The prediction of two short amphipathic α helices, respectively, consisting of residues 272 to 284 and 290 to 296 (fig. S2D), was corroborated by observations of spontaneous helix formation and membrane interaction in these regions during atomistic MD simulations of WIPI2d near the membrane, initially with an unstructured 6CD loop. In further simulations of the protein with the two predicted helices premodeled into the structure, spontaneous membrane insertion of residues ~271 to 300 was observed within 1.5 μs in one replicate (fig. S2E). The two putative PI(3)P binding sites (*28*, *30*, *44*) of WIPI2d, formed by blades 5 and 6 of the β-propeller, respectively, were also optimally positioned to bind PI(3)P in such a configuration (fig. S2E). These observations agree with previous studies suggesting amphipathic helix formation and membrane insertion of the 6CD region in the orthologous Autophagy-related 18 (Atg18) protein (*45*, *46*). Coarse-grained curvature-sampling simulations of WIPI2d 6CD residues 263 to 300 on buckled membranes yielded a mean local curvature of *H* ~ 0.015 nm^−1^ at the center of mass of the construct (fig. S2F), supporting its role as a curvature sensor, albeit with weaker curvature sensitivity (and stronger membrane binding) compared with helix α2 of ATG16L1 (Fig. 6E).

Previous work modeling membrane association of membrane curvature–generating proteins has highlighted the depth of helix insertion into the membrane as a key regulator of the magnitude of curvature sensitivity (*47*). We plotted the insertion depth of all-atom trajectories of WIPI2 loop and ATG16L1 α2 helix membrane insertion. In the membrane-bound state, the height *z* of the geometric center of the α2 helix backbone was located around the level of the phosphate groups. By contrast, the two short helices of the WIPI2d 6CD loop inserted deeper into the membrane by ~5 and ~20% of the thickness of the monolayer, respectively (fig. S3A). We observed the same trend in the insertion depths of α2 and 6CD in the coarse-grained simulation replicates (fig. S3B). Consistent with the membrane nanotube enrichment results, this finding supports a role for WIPI2 in strongly and stably associating with the membrane, whereas the ATG16L1 membrane insertion is responsible for weaker binding with heightened membrane curvature sensitivity.

## DISCUSSION

This study set out, in the first instance, to determine whether ATG12–ATG5-ATG16L1 bound to membranes in a curvature-dependent manner in the physiologically relevant setting of WIPI2-driven recruitment. We found that WIPI2 is itself curvature sensitive, but ATG12–ATG5-ATG16L1, following its recruitment by WIPI2, is even more sensitive. The sorting index for WIPI2-recruited ATG12–ATG5-ATG16L1 reaches a value of ~100 also seen for dedicated curvature sensors such as amphiphysin (*48*). MD simulations show that WIPI2 inserts substantially into the hydrocarbon core of the membrane, while helix α2 of ATG16L1 inserts less deeply. The comparatively shallow membrane insertion into the upper part of the lipid monolayer by amphipathic helices has been shown to correlate with high curvature sensitivity (*47*). Thus, the role of WIPI2 in this system is to drive membrane recruitment by binding tightly to PI(3)P and inserting deeply into the membrane, while ATG16L1 itself interacts weakly but in a highly curvature-dependent manner.

As noted previously (*26*), curvature dependence is stronger at a lower surface density. This likely represents the onset of saturation of the binding capacity of the membrane tube. It is also consistent with a model for curvature sensing in which self-association of ATG12–ATG5-ATG16L1 complexes with one another is neither required nor favorable. Curvature induction requires higher protein densities, at which the differential sorting of ATG12–ATG5-ATG16L1 to regions of high curvature is less evident.

There are some differences in the reported observations of ATG12–ATG5-ATG16L1 localization in cells between species. It is worth noting that ATG16L1 has almost negligible sequence similarity with yeast Atg16. The properties of yeast Atg16, which has been reported to tether (*49*) and deform (*50*) membranes, are therefore likely to differ from mammalian ATG16L1. In three-dimensional imaging of *Arabidopsis* cells, ATG5 is notably localized to toroidal zones that appear to correspond to the highly curved phagophore rim (*16*). In mammalian cells, however, two-dimensional electromagnetic imaging of thin sections in mouse embryonic stem cells suggested that ATG16L1 is uniformly localized on phagophores (*51*). Thus, the role of ATG12–ATG5-ATG16L1 in phagophore rim stabilization may vary in different organisms, cell types, and other conditions.

WIPI2 and ATG12–ATG5-ATG16L1 are exemplars of curvature-sensitive autophagy proteins whose curvature preference is strong enough that it could, in principle, contribute to stabilizing phagophore rim curvature. They are unlikely to be the only such proteins. The binding and/or activity of mammalian PI3KC3-C1 (*15*) and ATG3 (*17*) have been shown to be curvature sensitive in vitro. We found that ATG3 does not further increase the curvature sensitivity of ATG12–ATG5-ATG16L1; however, this is likely to reflect saturation of the already very high sorting index for ATG12–ATG5-ATG16L1, making it difficult to measure any further incremental increase. Saturation of the measurement may have led us to underestimate the true curvature dependence of ATG12–ATG5-ATG16L1 itself. It seems reasonable that both PI3KC3-C1 and ATG3 could contribute to phagophore rim stabilization. In vitro data are still lacking for ATG2A, but its apparent phagophore rim localization (*9*) makes ATG2A an intriguing candidate. Under curvature-inducing conditions, it might be expected that the rim would be saturated and broader localization of ATG2A would be observed. At any rate, further biophysical and cell biological characterization of the curvature dependence of PI3KC3 and ATG2A and comparison to the ATG12–ATG5-ATG16L1 data will be called for.

The data reported here are consistent with the previous report that ATG16L1 α2 is essential for LC3 lipidation and autophagosome formation (*18*). In that report, it was shown that recruitment of ATG16L1 to WIPI2 puncta, which mark sites of autophagy initiation, is unimpaired. Similarly, we found that GUV recruitment of the ATG12–ATG5-ATG16L1 FII mutant, which blocks membrane recruitment by α2, is unimpaired, because WIPI2 binding through the WIPI2-interacting region of ATG16L1 drives this process. The α2 mutant leaves intact the ATG12-5 unit, responsible for the recruitment of ATG3 and, in turn, for LC3 lipidation. Nevertheless, we found that mutation of helix α2 abolished LC3 lipidation activity in vitro, consistent with (*18*). We found that this was the case even in the presence of WIPI2 (fig. S4). Thus, ATG16L1 helix α2 has nonseparable roles in LC3 lipidation, directing ATG12–ATG5-ATG16L1 to sites of high curvature, and potentially in stabilizing the rim of the nascent phagophore.

## MATERIALS AND METHODS

### Protein expression and purification

ATG12–ATG5-ATG16L1-GFP constructs (Research Resource Identifier (RRID):Addgene_169077, Addgene_192705) were expressed and purified from Sf9 cells (American Type Culture Collection, catalog no. CRL-1711, RRID:CVCL_0549) as previously described (*25*) (dx.doi.org/10.17504/protocols.io.br6qm9dw). Briefly, cells were resuspended in lysis buffer [50 mM Hepes (pH 7.5), 300 mM NaCl, 2 mM TCEP (tris(2-carboxyethyl)phosphine), and cOmplete protease inhibitor (Roche)] and lysed by sonication. Lysate was clarified by centrifugation, and the soluble fraction was applied to a streptactin Sepharose column (Cytiva). Upon elution from the strep column with 5 mM desthiobiotin (Sigma-Aldrich), fractions were concentrated by filter centrifugation and purified by gel filtration over a Superose 6 column (Cytiva). mCherry-WIPI2d (RRID:Addgene_178912) was expressed and purified from suspension human embryonic kidney GnTI cells (*52*) (dx.doi.org/10.17504/protocols.io.bvjnn4me). Cells were resuspended in lysis buffer [50 mM Hepes (pH 7.5), 200 mM MgCl_2_, 10% glycerol, 1% Triton X-100, 1 mM TCEP, and cOmplete protease inhibitor] and lysed by gentle rocking at 4°C for 30 min. Lysate was clarified by centrifugation, applied to a streptactin Sepharose column, and eluted with buffer containing 10 mM desthiobiotin. GST-mCherry-FYVE (RRID:Addgene_192289, dx.doi.org/10.17504/protocols.io.ewov1n39pgr2/v1) and His-Tobacco Etch Virus protease (TEV)-ATG3 (RRID:Addgene_169079, dx.doi.org/10.17504/protocols.io.btgknjuw) were expressed and purified from *Escherichia coli* BL21(DE3) (New England Biolabs), catalog no. C2527) culture. LC3B (RRID:Addgene_190237, dx.doi.org/10.17504/protocols.io.j8nlkw82dl5r/v1) and ATG7 (https://dx.doi.org/10.17504/protocols.io.bsennbde) were purified as previously described.

### Protein labeling

ATG3 was labeled with ATTO 565 *N*-hydroxysuccinimide (NHS) ester (ATTO-TEC GmbH). Briefly, 40 μM ATG3 was mixed with 80 μM ATTO 565 NHS ester in 50 mM Hepes (pH 8.0), 150 mM NaCl, and 2 mM TCEP. The reaction was carried out for 1 hour at room temperature and buffer-exchanged over a G-25 desalting column (Cytiva) into 50 mM tris (pH 8.0), 150 mM NaCl, and 2 mM TCEP to quench the reaction and remove any unconjugated dye. Labeling efficiency was assessed by the ratio of absorbance at 280 and 564 nm, correcting for dye absorbance at 280 nm, using a Nanodrop spectrophotometer. (dx.doi.org/10.17504/protocols.io.x54v9d2zpg3e/v1).

### GUV preparation

GUVs were prepared by polyvinyl alcohol (PVA)–assisted swelling. Briefly, 100 μl of 5% PVA was spotted onto a glass coverslip and dried at 50°C. Fifty nanomoles of lipids dissolved in chloroform was mixed [mole percent: 70% 1,2-dioleoyl-sn-glycero-3-phosphocholine (DOPC), 20% 1,2,-dioleoyl-sn-glycero-3-phosphoethanolamine (DOPE), 5% 1,2,dioleoyl-sn-glycero-3-phospho-L-serine (DOPS), 5% 1,2-dioleoyl-sn-glycero-3-phospho-(1'-myo-inositol-3'-phosphate) (DO-PI(3)P), 0.3% ATTO647N-DOPE, and 0.01% 1,2-distearoyl-sn-glycero-3-phosphoethanolamine-N-[biotinyl(polyethylene glycol)-2000] (DSPE-PEG(2000) Biotin)] and dried on the PVA layer overnight in a vacuum desiccator. GUVs were swelled for 30 to 60 min at room temperature in 100 μl of sucrose solution slightly hypotonic to imaging buffer (320 mosM) as determined by the freezing point depression osmometer (Osmette III, Precision Systems). (dx.doi.org/10.17504/protocols.io.3byl4b398vo5/v1).

### Membrane tube assay

Proteins were mixed with fluorescently labeled GUVs and then added to a microscope chamber that had been passivated with bovine serum albumin (1 mg/ml) in imaging buffer and subsequently rinsed with imaging buffer [20 mM tris (pH 8.0), 150 mM NaCl, 2 mM MgCl_2_, and 2 mM TCEP]. GUVs were allowed to settle before adding streptavidin-coated silica beads (Spherotech) that had been diluted 1:1000 in imaging buffer. Using an optical trap, the bead was brought into contact with the biotinylated GUV surface and retracted to form a membrane tube. The protein bound to the GUV and tube membrane was monitored by confocal fluorescence imaging. (dx.doi.org/10.17504/protocols.io.ewov1n3dpgr2/v1).

### Imaging and image analysis

Imaging of the membrane tubes was performed on a Nikon Ti-Eclipse microscope with a Nikon A1 confocal unit, modified with an optical trap and micromanipulators (*53*, *54*), using a Plan Apochromat 60×/1.20 water immersion objective (Nikon). A complete image dataset for this paper can be found at 10.5281/zenodo.6508734. Image files were processed in ImageJ (RRID:SCR_003070, https://imagej.nih.gov/ij/, RRID:SCR_003070) to generate regions of interest (ROIs) containing segments of the membrane tube or of the GUV surface. ROIs were combined and analyzed using custom Python scripts (doi.org/10.5281/zenodo.7058704). Briefly, the ROIs were segmented on the basis of the intensity of the membrane dye channel with an Otsu threshold for local maxima, and protein binding signal was quantified as the average value in the protein label channel masked with the membrane channel segmentation. Background was calculated as the average value of pixels not in the membrane channel mask and subtracted from the signal as calculated above. Protein enrichment on membrane tubes (*S*) was calculated as a ratio of protein intensity on the tube to protein intensity on the GUV surface, normalized for membrane intensity

$$S=\frac{\frac{I_{\;\mathrm{prot},\mathrm{tube}}}{I_{\mathrm{mem},\mathrm{tube}}}}{\frac{I_{\;\mathrm{prot},\mathrm{GUV}}}{I_{\mathrm{mem},\mathrm{GUV}}}}$$

where *I*_prot_ and *I*_mem_ are the average intensity values from the ROI for the protein or membrane fluorescence channel, on the tube or GUV surface (dx.doi.org/10.17504/protocols.io.5jyl891e9v2w/v1).

### Tube radius calculation

Tube radii were calculated using a previously described method (*55*) in which the ratio of membrane dye fluorescence in the tube ROI to that in the GUV ROI is multiplied by an experimentally derived calibration constant (*k*_tub_)

$$R=k_{\mathrm{tub}}\left(\frac{I_{\mathrm{mem},\mathrm{tube}}}{I_{\mathrm{mem},\mathrm{GUV}}}\right)$$

where *R* is the tube radius, and *I*_mem,tube_ and *I*_mem,GUV_ are the average intensity values of the membrane dye from the tube or GUV ROI.

To determine the value of *k*_tub_, tubes were pulled from GUVs held on an aspiration pipette, and the membrane tension varied by changing the aspiration force with a microfluidic controller (MFCS-EZ, Fluigent) to generate tubes of varying radii. Membrane tension (σ) was determined from aspiration pressure and microscopy images as

$$\sigma =\Delta P\frac{r_{\;\mathrm{pip}}}{2\left(1-\frac{r_{\;\mathrm{pip}}}{r_{\mathrm{GUV}}}\right)}$$

where ∆*P* is the difference in aspiration pressure from baseline, and *r*_pip_ and *r*_GUV_ are the radii of the aspiration pipette and the GUV, respectively.

Tube radius was subsequently calculated from membrane tension and force (*F*) felt by the bead in the optical trap as

$$R=\frac{F}{4\pi \sigma }$$

and plotted against the ratio of tube to GUV membrane fluorescence (fig. S1B). Then, *k*_tub_ was extracted from the slope of a linear least squares regression (dx.doi.org/10.17504/protocols.io.x54v9y3rqg3e/v1).

### Surface density calculation

Protein density on the GUV surface was calculated by drawing a relationship between known concentrations of protein and membrane label in bulk solution and on the GUV surface (*55*). First, standard curves of GFP and DOPE-ATTO488 were generated from a serial dilution in imaging buffer and 0.1% Triton X-100. The ratio of the slopes of the linear fits was used to correct for differences in optical properties of the different fluorophores. Then, the fluorescence intensity of GUVs containing a range of known percentages of dye-conjugated lipid (0.01 to 1 mole percent) was calculated and plotted against surface density of the dye-conjugated lipid (assuming 0.7 nm^2^ per lipid) to give a relationship between intensity and GUV surface density. This was corrected by the ratio of intensities of the fluorophores in bulk solution (dx.doi.org/10.17504/protocols.io.81wgb65nnlpk/v1).

### LC3 lipidation assay

LC3B in vitro lipidation reaction was carried out as in (*25*). Liposomes extruded to 400 nm from a lipid suspension [1 mg/ml; 70% DOPC, 20% DOPE, 5% DOPS, and 5% PI(3)P] were mixed 1:1 with purified lipidation components to a final concentration of 1 μM ATG3, 1 μM ATG7, 500 nM WIPI2, 100 nM ATG12–5-16L1–GFP, and 2.5 μM LC3B in a reaction buffer containing 20 mM tris (pH 8.0), 150 mM NaCl, 1 mM MgCl_2_, 1 mM TCEP, and 1 mM adenosine 5′-triphosphate. The reaction mix was incubated at 37°C, and fractions were removed at 0, 15, 30, 60, and 120 min and quenched by adding 1× SDS loading buffer and heating to 60°C for 10 min. LC3 lipidation was assessed by Coomassie-stained SDS–polyacrylamide gel electrophoresis. (dx.doi.org/10.17504/protocols.io.kqdg392ypg25/v1)

### MD simulations

MD simulations were performed with GROMACS 2020 (RRID:SCR_014565, https://manual.gromacs.org/documentation/2020/index.html)(*56*), using the CHARMM36m force field (RRID:SCR_014892)(*57*) for all-atom simulations and the MARTINI 3 force field (RRID:SCR_021951, http://cgmartini.nl/index.php/martini-3-tutorials) (*58*) and Gō-MARTINI model (*59*) for coarse-grained systems. Atomistic models of the ATG12–ATG5-ATG16L1 and WIPI2d-ATG16L1 complexes were based on crystal structures with Protein Data Bank (PDB) IDs 4NAW (*60*) and 7MU2 (*23*), respectively. The ATG16L1 N-terminal helix in the former complex was replaced by a more complete structure [PDB ID: 4TQ0; (*61*)] and residues 1 to 9 were added using the DEMO server (https://zhanggroup.org/DEMO/) (*62*) to give a model of residues 1 to 50. A second model of the same region was generated in PyMOL (RRID:SCR:000305, https://pymol.org/) (*63*) by rotation of helix α2 relative to α1 at the Gln^30^/Ala^31^ hinge. For WIPI2d, residues 262 to 299 of the 6CD region were modeled either (i) as an unstructured loop using SWISS-MODEL (RRID:SCR_018123, https://swissmodel.expasy.org/) (*64*) or (ii) with two short helices based on the structure predicted by AlphaFold (https://alphafold.ebi.ac.uk/) (*65*, *66*), in two alternative models. Unstructured ATG12 residues 1 to 52 and WIPI2d residues 1 to 11 and 362 to 425 were excluded from the models. The Lys^130^ side chain of ATG5 was connected to the backbone carbonyl of ATG12 Gly^140^ by an isopeptide bond. Exposed N- or C-terminal groups at the end(s) of each incomplete structure or truncated construct were neutralized. His^183^ and His^255^ at the putative PI(3)P binding sites of WIPI2d were protonated.

All membranes were prepared initially in a coarse-grained representation using the insane method (*67*) and consisted of 60% DOPC, 20% DOPE, 5% DOPS, 10% POPI (1-palmitoyl-2- oleoyl-sn-glycero-3-phosphoinositol), and 5% PI(3)P based on the endoplasmic reticulum lipid composition (*68*). Buckled membranes were constructed using LipidWrapper (*70*) (https://git.durrantlab.pitt.edu/jdurrant/lipidwrapper) (*69*) by fitting the height (amplitude) of the membrane as a sine function of its *x* coordinate. For all-atom simulations, the CG2AT2 tool (*70*) (https://github.com/owenvickery/cg2at) was used to convert each equilibrated membrane system to an atomistic representation. All simulation systems were solvated with 150 mM aqueous NaCl, using TIP3P (transferable intermolecular potential with 3 points) or coarse-grained water. Atomistic models of ATG12–ATG5-ATG16L1 or WIPI2d were placed above membranes after CG2AT2 conversion (upon removal of coarse-grained solvent). In the case of the remodeled ATG16L1, the embedded helix α2 configuration was obtained by insertion into either leaflet of the buckled membrane at the coarse-grained stage, with all particles of the helix around or below the level of phosphate particles and the hydrophobic face oriented toward the membrane core. Lipids clashing with the embedded helix were removed from both leaflets, followed by protein-membrane equilibration and CG2AT2 conversion of the system as described above. The CG2AT2 “align” option was used, with the initial atomistic ATG12–ATG5-ATG16L1 complex containing the remodeled ATG16L1 (in its conformation before simulation) aligned to and replacing the back-mapped protein in the CG2AT output for subsequent equilibration. Simulation replicates were independently prepared and equilibrated, with the simulation cells having approximate dimensions of 14 nm by 14 nm by 20 nm for atomistic WIPI2d simulations, 32 nm by 14 nm by 28 nm for simulations of atomistic ATG12–ATG5-ATG16L1 on either side of curved membranes (with two copies per replicate system), and 63 nm by 28 nm by 38 nm for coarse-grained curvature-sampling simulations. The *xy* dimensions of buckled membrane systems were fixed during simulation.

Each coarse-grained membrane system was equilibrated for 200 ns, with atomistic systems further equilibrated for 10 ns upon conversion from coarse-grained representation. Harmonic positional restraints were applied to nonhydrogen protein atoms or backbone beads during equilibration, with a force constant of 1000 kJ mol^−1^. In the case of buckled membranes, a weaker (10 kJ mol^−1^) restraint in *z* was also applied to the phosphorus atoms or phosphate beads of lipid headgroups to preserve the initial distance between protein and membrane at the equilibration stage. System temperature and pressure were maintained at 310 K and 1 bar, using the velocity-rescaling thermostat (*71*) and a semi-isotropic Parrinello-Rahman barostat (*72*) during the production phase. Atomistic and coarse-grained systems were simulated with integration time steps of 2 and 20 fs, respectively. For the atomistic simulations, long-range electrostatic interactions were treated using the smooth particle mesh Ewald method (*73*, *74*) with a real-space cutoff of 1 nm, a Fourier spacing of 0.12 nm, and charge interpolation through fourth-order B splines. The linear constraint solver (LINCS) algorithm was used to constrain covalent bonds involving hydrogen atoms (*75*).

Simulation trajectories were analyzed through MDAnalysis 2.0 (https://www.mdanalysis.org/2021/08/22/release-2.0.0/)(*76*, *77*) in Python 3.6 (RRID:SCR_008394, https://www.python.org/downloads/release/python-360/). The local curvature of buckled membranes during simulation was estimated using the MemCurv software package (https://github.com/bio-phys/MemCurv) version 1.0 following the established protocol and parameter settings (*41*). Sampling at 10-ns intervals, membrane profiles were approximated using a two-dimensional Fourier expansion and optimized by least-squares fitting. The mean curvature *H* at any given position of interest was derived from the shape operator of the approximated profile along the membrane surface (*41*).

## Notes

View/request a protocol for this paper from *Bio-protocol*.
