# Supplementary material for: Membrane curvature sensing and stabilization by the autophagic LC3 lipidation machinery
Source: Sci Adv. 2022 Dec 14;8(50):eadd1436. doi: 10.1126/sciadv.add1436 (PMC9750143; doi:10.1126/sciadv.add1436)
Supplement: Supplementary file 1 — Figs. S1 to S5 [file sciadv.add1436_sm.pdf]

Science Advances
AAAS

Supplementary Materials for

Membrane curvature sensing and stabilization by the autophagic LC3 lipidation machinery

Liv E. Jensen *et al.*

Corresponding author: James H. Hurley, jimhurley@berkeley.edu

Sci. Adv. **8**, eadd1436 (2022)
DOI: 10.1126/sciadv.add1436

This PDF file includes:

Figs. S1 to S5

Supplementary Materials

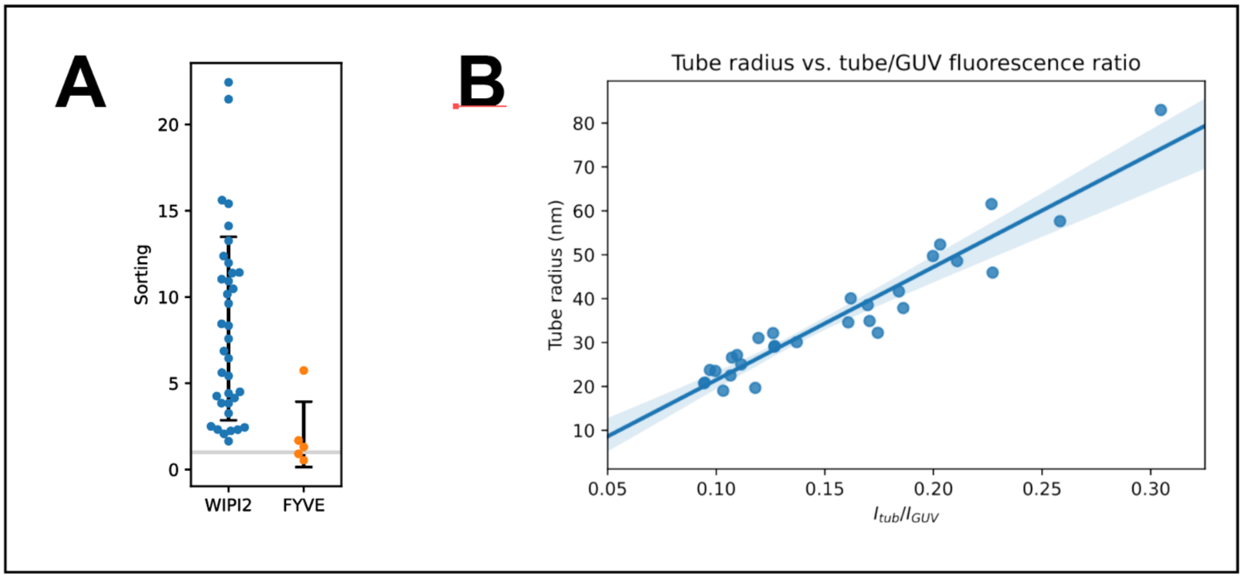

Fig. S1. Membrane tube sorting and curvature quantification. (**A**) Swarm plot comparison of WIPI2 (blue) compared to FYVE (orange) sorting on membrane tubes. Black bars indicate standard deviation; grey horizontal line at S=1. (**B**) Tube radius calculated from optical trap force and GUV aspiration pressure vs. ratio of fluorescence intensity of membrane tube to GUV surface. Linear regression plotted as a solid blue line. Light blue shaded region depicts a 95% confidence interval.

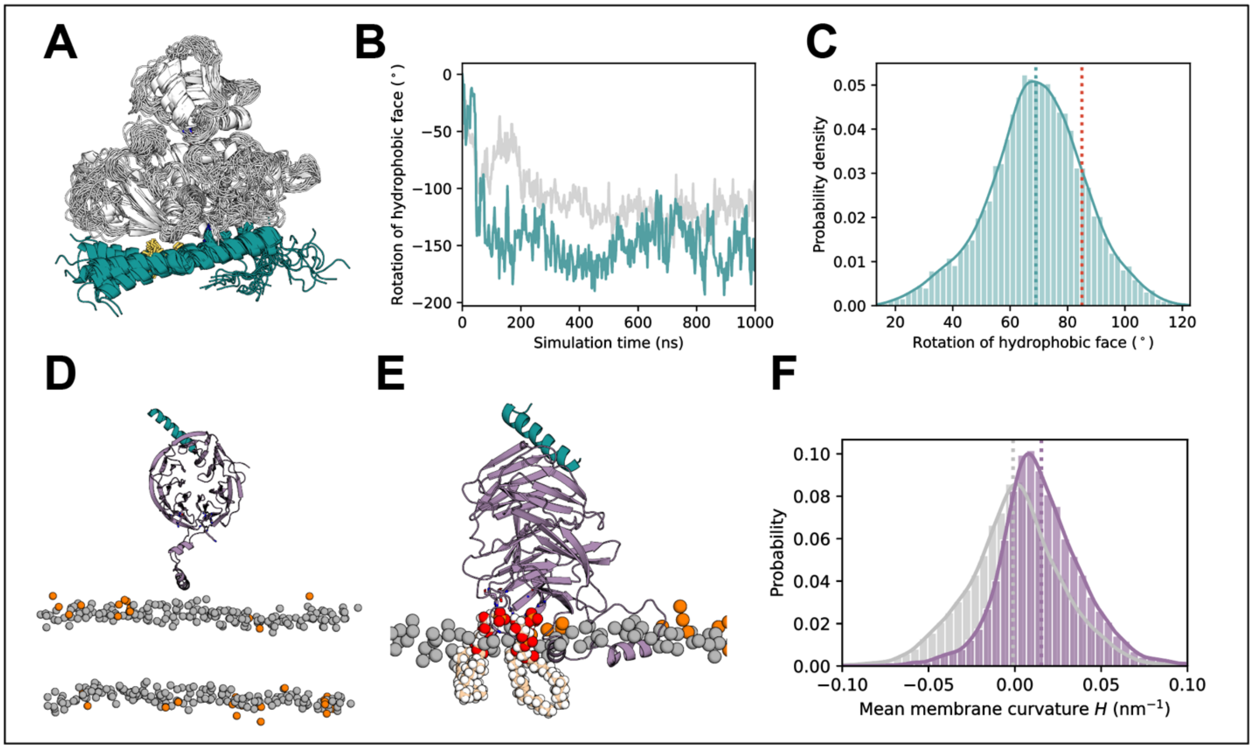

Fig. S2. Membrane binding and curvature sensing of ATG12-5–L1 and WIPI2d. (**A**) Flexibility of ATG16L1 helix α2, illustrated with a superimposition of ATG12-5–L1 conformations sampled at 50 ns intervals during one 1 µs simulation trajectory. The side chains of ATG16L1 helix α1 residues Ile17, Leu21, and Arg24 (cyan) at the interface with ATG5, and of helix α2 residues Phe32, Ile35, and Ile36 of the hydrophobic face (highlighted yellow) are shown in stick representation. (**B**) Reorientation of the helix α2 hydrophobic face of the aforementioned molecule (cyan) over the course of its simulation, and of a second copy of ATG12-5–L1 (grey) on the opposite membrane leaflet in the same replicate system. At each time point, the rotation angle (relative to the initial conformation) is defined by taking the outward-pointing normal to the plane through the Cα atoms of Phe32, Ile35, and Ile36. Rotation in the clockwise direction, as viewed from the C-terminal end of helix α2 corresponds to a positive value. (**C**) Distribution of helix α2 hydrophobic face rotation angles in simulations of a remodeled ATG16L1 structure in complex with ATG12-5, over a total simulation time of ~1.8 µs. Rotation angles are measured relative to the conformation in the crystal structure (PDB ID: 4NAW). The remodeled configuration had an initial value approximately in the –*z* direction into the membrane, as indicated by the red dotted line. The mean position of the distribution is indicated by the cyan dotted line. (**D**) Structure of WIPI2d (magenta) bound to residues 209-230 of ATG16L1 (PDB ID: 7MU2), with the WIPI2d 6CD residues 272-284 and 290-296 modelled as α-helices, placed above a PI(3)P-containing membrane. Residues forming the two putative PI(3)P binding sites in blades 5 and 6 are shown in stick representation. Phosphorus atoms of PI(3)P molecules are shown as orange spheres, with those of all other lipid headgroups colored grey. (**E**) Snapshot of WIPI2d after forming spontaneous membrane interactions during one (2 µs) simulation replicate, with residues ~271-300 embedded into the membrane. Two PI(3)P molecules, each occupying a binding site on WIPI2d, are highlighted as spheres. (**F**) Probability histogram of local mean curvature values sampled at the center of mass of WIPI2d 6CD residues 263-300 (magenta) and at random lipid phosphate positions (grey) during the same simulations, with data collected over the final 12 µs of six independent 20 µs replicate systems, each containing two copies of the protein construct, on either side of the buckled membrane.

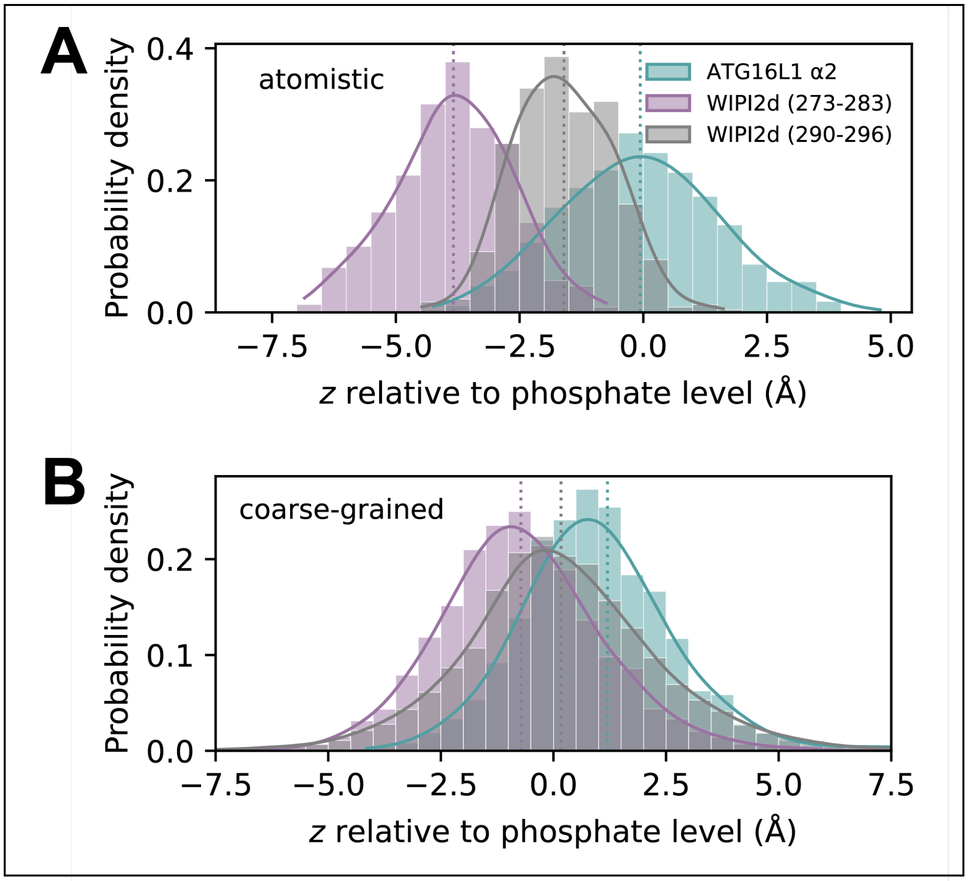

Fig. S3Membrane insertion depths of amphipathic helical elements within WIPI2d and ATG16L1. (**A**) The *z*-positions of the backbone center of geometry of WIPI2d residues 273-283 (around a mean value of ~−3.8 Å) and 290-296 (~−1.6 Å) and of ATG16L1 helix α2 are each measured relative to the level of phosphate groups (taking the center of mass of each phosphorus atom with its bound oxygens) of lipids within 4 Å of the helix in the membrane leaflet to which the protein is bound. The distribution of *z*-positions across all-atom simulation replicates of ATG12–ATG5-ATG16L1 (six 300 ns trajectories) and over the all-atom WIPI2d simulation where spontaneous insertion occurred (one 2 µs replicate) is shown, respectively. Simulation trajectories are sampled at 1 ns intervals after an equilibration period following membrane insertion of the amphipathic helix. (**B**) Corresponding insertion depth measurements taken from coarse-grained simulations of WIPI2d 6CD and ATG16L1 helix α2 constructs (residues 263-300 and 26-45, respectively), each across six 50 µs replicates. The mean *z*-position of phosphate beads of lipids within 6 Å of the embedded helix is sampled at 0.1 µs intervals.

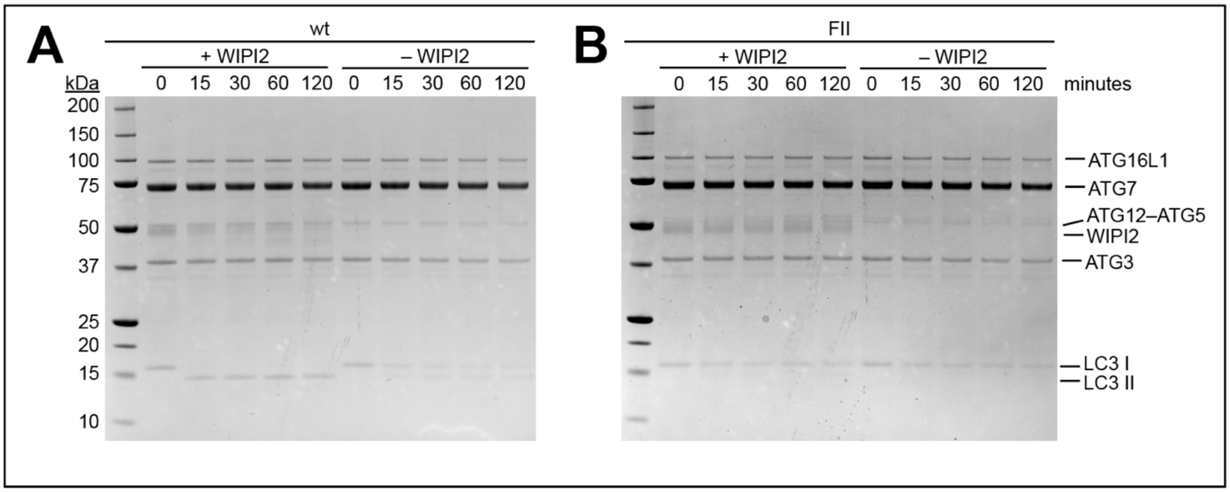

Fig. S4 LC3 lipidation with ATG16L1 helix α2 mutant. SDS-PAGE gel of *in vitro* LC3 lipidation reaction on 400nm extruded liposomes containing wt (**A**) or FII (**B**) ATG16L1-GFP. Liposomes were incubated with 1μM ATG3, 1μM ATG7, 100nM ATG12–5-16L1, 2.5μM LC3B, 1mM ATP in the presence or absence of 500nM WIPI2. Timepoints were collected at 0, 15, 30, 60, and 120 minutes incubation at 37˚C.

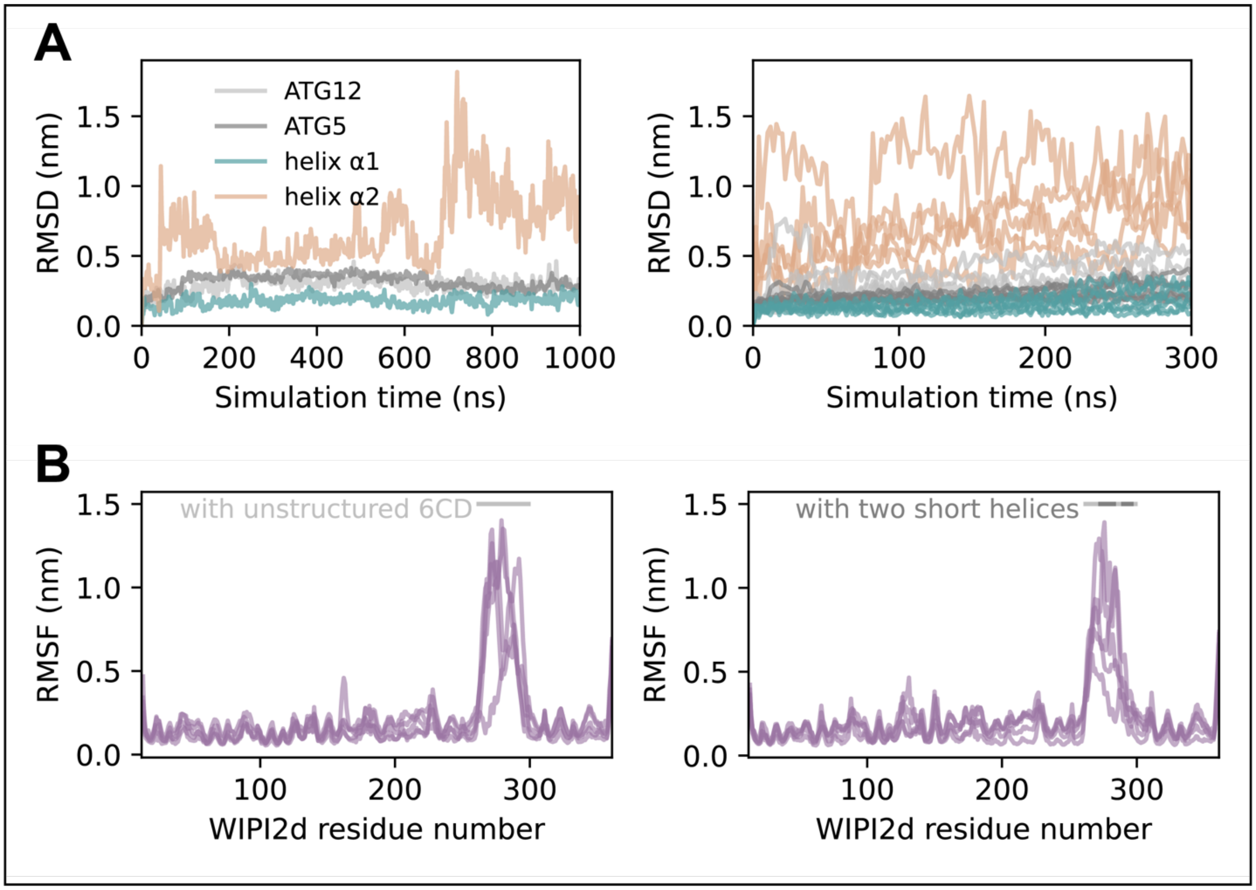

Fig. S5 Root-mean-square deviation (RMSD) and root-mean-square fluctuation (RMSF) assessment of protein stability. (**A**) Cα RMSDs of the ATG12–ATG5-ATG16L1 complex during a 1 μs simulation trajectory with considerable reorientation of helix α2 (left) and over 300 ns simulation replicates using the remodeled ATG16L1 structure (right). In each case, the Cα RMSD was calculated upon least-squares fitting to the initial ATG12–ATG5-α1 conformation. (**B**) Cα RMSFs of WIPI2d residues during five 300 ns simulation replicates of the protein modeled with an unstructured 6CD loop (left) and during five 1-2 μs replicates using a structure containing pre-modeled helices in the 6CD region (right). The location of the 6CD loop along the WIPI2d sequence is indicated with a horizontal line.
